# Time-restricted feeding suppresses excess sucrose-induced plasma and liver lipid accumulation in rats

**DOI:** 10.1371/journal.pone.0201261

**Published:** 2018-08-15

**Authors:** Shumin Sun, Fumiaki Hanzawa, Miki Umeki, Saiko Ikeda, Satoshi Mochizuki, Hiroaki Oda

**Affiliations:** 1 Laboratory of Nutritional Biochemistry, Nagoya University, Nagoya, Japan; 2 Department of Nutritional Science, Nagoya University of Arts and Sciences, Nisshin, Japan; 3 Faculty of Education and Welfare Science, Oita University, Oita, Japan; Universidade do Estado do Rio de Janeiro, BRAZIL

## Abstract

The etiology of metabolic syndrome involves several complicated factors. One of the main factors contributing to metabolic syndrome has been proposed to be excessive intake of sucrose, which disturbs hepatic lipid metabolism, resulting in fatty liver. However, the mechanism by which sucrose induces fatty liver remains to be elucidated. Considering feeding behavior important for metabolism, we investigated whether time-restricted feeding of high sucrose diet (HSD), only in the active phase (the dark phase of the daily light/dark cycle), would ameliorate adverse effects of sucrose on lipid homeostasis in rats. Male Wistar rats, fed either an *ad libitum (ad lib*.*)* or time-restricted control starch diet (CD) or HSD were investigated. Rats fed *ad lib*. (CD and HSD) completed approximately 20% of food intake in the daytime. Time-restricted feeding did not significantly suppress total food intake of rats. However, time-restricted feeding of HSD significantly suppressed the increased plasma triglyceride levels. Moreover, time-restricted feeding also ameliorated HSD-induced liver lipid accumulation, whereas circadian oscillations of liver clock gene or transcriptional factor gene expression for lipid metabolism were not altered significantly. These results demonstrated that restricting sucrose intake only during the active phase in rats ameliorates the abnormal lipid metabolism caused by excess sucrose intake.

## Introduction

Metabolic syndrome is a cluster of symptoms, including type 2 diabetes, hypertension, hyperlipidemia, and central obesity, without internationally agreed definition[1]. And nowadays it is a global problem threatening human health. The etiology of metabolic syndrome is quite complicated owing to the involvement of both genetic and environmental factors. Among the environmental factors, diet plays a significant role in the development of metabolic syndrome [2]. Excessive energy from a high-fat diet was considered to be the main factor of metabolic syndrome. Moreover, excess sucrose intake has also been recognized as a culprit recently [3]. Fructose, a monosaccharide of sucrose hydrolysis, was concluded to play an important role in the pathogenesis of metabolic diseases, including non-alcoholic fatty liver and hyperlipidemia [4,5]. As reported, glucose is metabolized through the glycolysis pathway, which is controlled by several factors such as adenosine triphosphate (ATP) and citrate [6]. Moreover, the high Michaelis constant (Km) of glucokinase, a rate-limiting enzyme regulated by insulin, contributes to the regulation of glucose metabolism [7]. Remarkably distinct from this, fructokinase, the first enzyme metabolizing fructose, has a low Km [8]. This results in a series of rapid substrates for the hepatic *de novo* lipogenesis [9,10]. However, based on the current studies that primarily focus on the flow of substrates from fructose into fatty acid synthesis, the relationship between sucrose and metabolic syndrome remains controversial.

Further studies are required to uncover the mechanism of how sucrose could contribute to the onset of metabolic syndrome at the molecular level. At present, efficient prevention or amelioration methods for sucrose-induced metabolic syndrome are required. We had previously demonstrated that a suppressed feeding schedule, with high-cholesterol diets every 6 hr, results in rats developing hypercholesterolemia and a disturbed liver clock [11]. Disruption of circadian oscillations in *Clock* mutant mice leads to robust attenuation of feeding rhythm and metabolic syndrome [12]. Moreover, time-restricted feeding of a high-fat diet for 8 hr per day in mice improved metabolic rhythm and imparted protection from metabolic diseases, thereby highlighting the importance of both nutrient state and feeding pattern in metabolic homeostasis [13]. Also, the beneficial effects of time-restricted feeding regimen in different diet conditions, including high-fat, high-fructose, and high-fat-plus-high-fructose diets, have been verified in mouse models [14].

Time-restricted feeding regimen in Zucker rat models of obesity, with normal chow diet, succeeded to reduce body weight gain [15]. Here, in order to evaluate the effects of time-restricted feeding on amelioration of sucrose-induced metabolic syndrome in genetically normal rats, such as abnormal lipid metabolism, we established a feeding schedule by restricting high sucrose diet (HSD) to the 12 hr long active phase of male Wistar rats. Rats may be more appropriate animal models than mice to investigate metabolic diseases of humans, due to their approximately 10-times larger body size and more stable energy metabolism statuses compared to that in mice. We found that time-restricted feeding of HSD suppressed the excess sucrose-induced lipid accumulation effectively both in blood and liver, when compared with rats fed HSD *ad lib*., without significantly altering the circadian oscillations of clock gene expression in liver.

## Materials and methods

### Animals, feeding schedule, and diets

**Fig 1:**
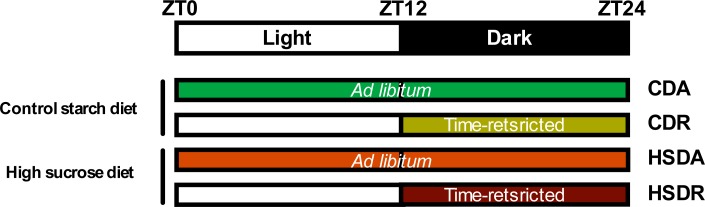
Schematic illustration of four feeding regimens. ZT: zeitigeber time.

The rodent study was approved by and performed according to the guidelines of the Animal Research Committee of the Center for Animal Research and Education, Nagoya University (Permit Number: 2015052901). All surgeries were performed under isoflurane anesthesia, and all efforts were made to minimize suffering. Five-week-old Wistar male rats (Japan SLC, Japan), weighing about 90g each, were independently housed under a 12 hr light: 12 hr dark cycle (lights on 0800–2000) at a temperature of 23±1°C; free access to water was provided throughout the experiment. They were allowed to adapt to the housing conditions for 3 d following which temperature data loggers (KN laboratories, Osaka, Japan) were implanted into the intraperitoneal cavity. Body weight of rats was measured at ZT2 (Zeitgeber time, ZT0 is defined as the point of lights on) everyday. After 3 d, they were divided into four groups, with both equivalent initial body weights and plasma triglyceride concentrations. Rats were fed the experimental diets for four weeks. The compositions of diets are shown in Table 1. They were fed control starch diet (CD) or high sucrose diet (HSD) either with *ad lib*. (named as group CDA or HSDA, respectively) or time-restricted (named as group CDR or HSDR, respectively) access to diets (see Fig 1). Under time-restricted feeding regimen, rats were allowed to access the diets during the active phase (dark period) between ZT12 and ZT24. Rats were kept in wire-bottom cages individually to avoid coprophagy and were fed powder diets from containers. During non-feeding phases, the containers were moved out of the cages to ensure no food left. Blood sampling from rat caudal vein, using heparin-treated capillaries, was performed at ZT5 after 4 hr fasting on days 0 (the start of experimental diets), 1, 3, 7, 14, and 24. Plasma was prepared by centrifuging samples at 1500 × g (4°C) for 10 min; blood supernatant fraction collected with heparin treatment (as mentioned above) was named as plasma. Rats were sacrificed by decapitation without anesthesia at ZT2, ZT8, ZT14, ZT18, and ZT22 from day 28 to day 29. When rats were sacrificed at night (ZT14, 18, and 22), they were exposed to light only at the moment of decapitation, to minimize the influence of light. Blood was collected without heparin-treatment and serum was prepared by centrifuging samples at 1500 × g (4°C) for 10 min. Livers and epididymal adipose tissues were excised and frozen immediately in liquid nitrogen and stored at -80°C until further analysis.

**Table 1:** Compositions of the experimental diets.

Ingredient, g/kg diet	Control starch diet (CD)	High sucrose diet (HSD)
**Starch**	653	-
**Sucrose**	-	653
**Casein**	200	200
**Mineral mixture**^1^	35	35
**Vitamin mixture**^2^	10	10
**Cholin chloride**	2	2
**Cellulose**	50	50
**Corn oil**	50	50

^1^AIN-93G-MX

^2^AIN-93VX

### Biochemical analysis

The blood triglyceride, cholesterol, glucose, non-esterified fatty acid (NEFA) and bile acid concentrations were measured using commercial kits (triglyceride E-test, T-cholesterol E-test, glucose CII-test, NEFA-C test and TBA-test; Wako Pure Chemical Industries, Osaka, Japan). The insulin and corticosterone levels were determined using ELISA kits (rat insulin ELISA kit, Morinaga Institute of Biological Science, Yokohama, Japan; corticosterone ELISA kit, Enzo life sciences, NY).

About 2.5 g of liver was homogenized and lipids extracted, following the method described by Folch *et al*. [16]. Total lipids in the liver were determined gravimetrically. The concentrations of hepatic triglycerides, cholesterol, and phospholipid in the extracts were measured using commercial kits (phospholipids C-test: Wako Pure Chemical Industries, Osaka, Japan).

### Gene expression analysis

Total RNA was extracted from 500 mg liver tissue of each rat, following the method of Chomczynski and Sacchi [17]. RNA quality was confirmed by northern blotting. Deoxyribonuclease (DNase) treatment was performed with ribonuclease (RNase)-free DNase (Promega, Wisconsin) and RNase inhibitor (Takara Bio, Japan). Complementary DNA (cDNA) was synthesized with 2 μg DNase-treated RNA by reverse transcription kit (High capacity RNA-to-cDNA kit, Applied Biosystems, CA). The cDNA was used to determine messenger RNA (mRNA) levels by quantitative real-time polymerase chain reaction (real-time PCR) analysis. The *APOE* mRNA level was not affected by any treatment in this study and was used as the normalization standard. Sequences of the primer sets used are shown in S1 Table.

### Body temperature analysis

Rat body temperatures were recorded every 10 min from day 0 to the end of the experiment. Rh Manager program (KN laboratories, Osaka, Japan) was used to analyze body temperature variations. Although variations were monitored every 10 min, those every 90 min were depicted in results.

### Statistics

At the end of the experiment, samples were collected throughout the day to evaluate the diurnal changes of serum parameters and hepatic gene expressions. Data displayed in tables and figures represent mean ± standard error of mean (SEM). Body weight gain, food intake, liver weight, epididymal adipose tissue weight, liver lipids, serum parameters, and real-time PCR results were analyzed with two-way ANOVA. Plasma parameters were analyzed with two-way repeated measures ANOVA. For body temperature variations, two-way repeated measures ANOVA was followed by JTK_CYCLE analysis [18] to determine the phases and amplitudes in each rat. Finally, two-way ANOVA was performed to analyze phases and amplitudes among four groups. Percentage of food intake in light (or dark) period was analyzed by Student’s *t* test. Statistical results are presented in Table 2 and S2–S6 Tables. All statistical analyses were performed with IBM SPSS Statistics (Version 22) and R studio.

**Table 2:** Body weight gain, food intake and tissue weights of rats.

	CDA	CDR	HSDA	HSDR	Two-way ANOVA		
					Diet-effect	Timing-effect	Interaction
**Body weight gain, g/28d**	137.06±2.73	124.77±3.19 ***	134.11±2.77	133.56±2.66	NS	0.05	0.05
**Food intake of d18-20, g**	49.26±0.85	47.04±0.93	47.85±1.01	49.05±1.11	NS	NS	NS
**Liver weight, g/100g body weight**	3.84±0.05	3.85±0.07	4.45±0.08	4.45±0.10	0.05	NS	NS
**Epididymal adipose tissue weight, g/100g body weight**	1.91±0.05	1.77±0.05	2.02±0.08	1.92±0.08	NS	NS	NS

Each data represents mean ± SEM, n = 20. CDA: control starch diet *ad lib*.; CDR: control starch diet time-restricted; HSDA: high sucrose diet *ad lib*.; HSDR: high sucrose diet time-restricted; 0.05 represents for significant changes; NS: not significant. When the interaction is significant, Student’s *t* test using the residual mean square was performed.

*** indicated the value differed significantly (p<0.001) from the value of CDA group.

## Results

### Body weight, food intake, and tissue weights

**Fig 2:**
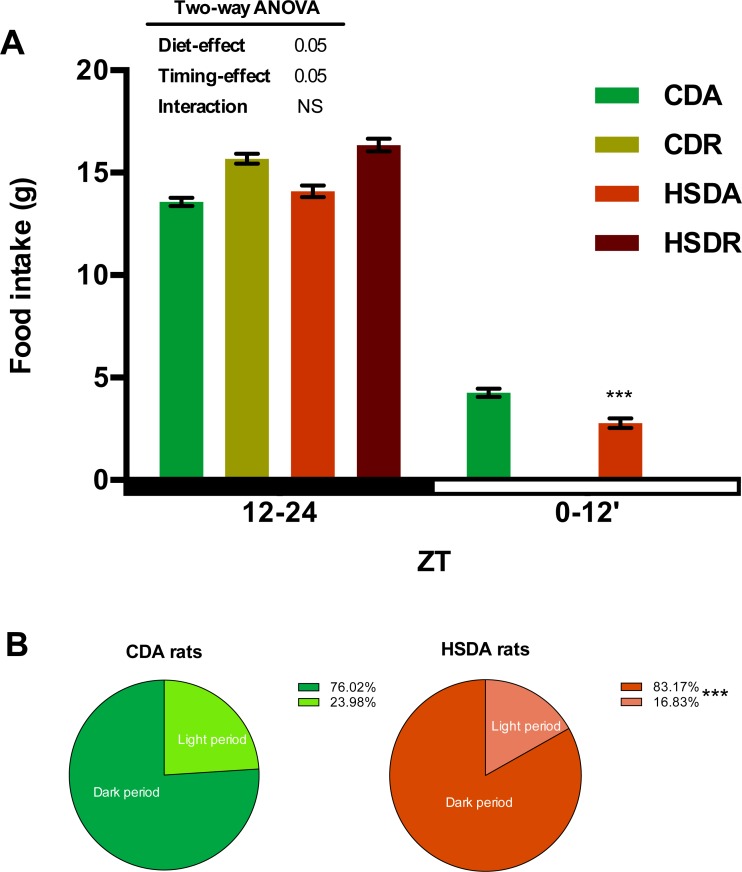
HSD altered feeding behavior in rats. (A) Food intake in light/dark period was respectively measured from d18 to d20 and time-restricted feeding rats did not access to food during light periods. Values are means ± SEM, n = 20. Food intake of dark period was analyzed by two-way ANOVA. 0.05 represents for significant changes and NS means not significant. Food intake of light period was analyzed by Student’s *t* test and *** indicated the value differed significantly (p<0.001) from the value of CDA group. (B) Rats fed a HSD had less food intake in dark period. The total amount of food intake in a whole day is defined as 100%. Values are means, n = 20. *** indicated the value differed significantly (p<0.001) from the value of CDA group by Student’s *t* test.

Body weight gain had no obvious change among CDA, HSDA, and HSDR groups, while group CDR showed lowered body weight gain (Table 2). Total food intake did not change among the four groups (Table 2). However an altered day/night food intake ratio between CDA and HSDA groups was observed. Since rats are nocturnal animals, most of their food intake is during the dark period (ZT12-ZT24) (Fig 2A). We measured the light-dark period food intake ratio of groups fed *ad lib*. (Fig 2). Although HSDA rats showed approximately similar food intake as CDA rats (Table 2), the latter completed 24% of their daily food intake during the inactive phase (light period), whereas HSDA rats had only 17% at that time (Fig 2B). This result indicated a potential role of HSD in altering the diurnal feeding rhythm in rats. The total food intake showed no significant difference between time-restricted feeding and *ad lib*. feeding rats (Table 2), hence indicating that the feeding regimen did not change the amount of food intake. Rats fed an HSD, either *ad lib*. or as time-restricted, displayed obviously increased liver weights (Table 2). However, epididymal adipose tissue weights were not different among the groups (Table 2).

### Body temperatures

**Fig 3:**
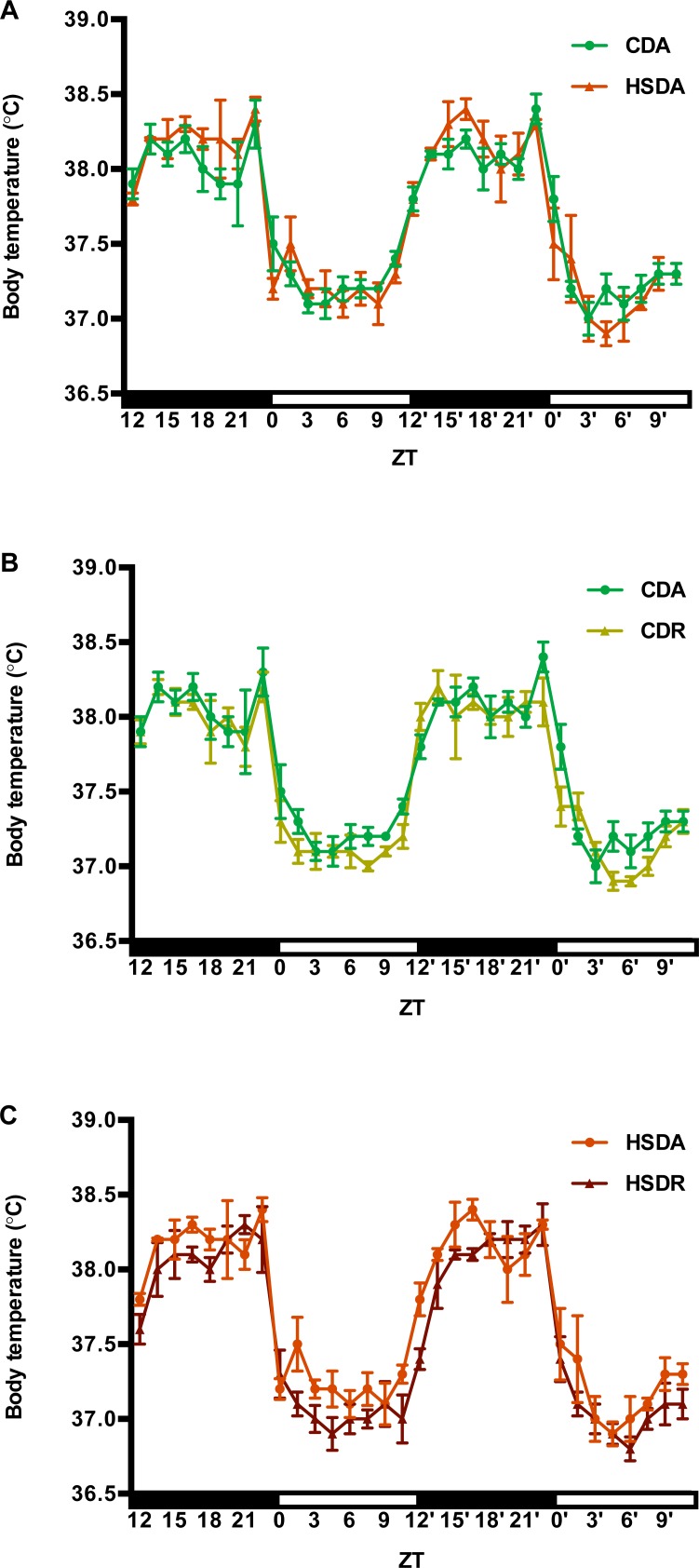
Time-restricted feeding of HSD affected the oscillation pattern of body temperatures in rats. Body temperatures were recorded every 10 min during the whole 4-week study, and averages of every 90 min in the final two experimental days were figured here. Values are means ± SEM. Two-way repeated measures ANOVA of body temperature between every two groups and also among 4 groups, were firstly performed. JTK_cycle analysis was applied to determine the phases and amplitude of body temperatures in each rat (mean ± SEM of each group was shown in S2 Table). Then, two-way ANOVA was performed to analyze phase and amplitudes among four groups. Statistical results of JTK_cycle and two-way ANOVA are present in S2 Table. 0.05 represents for significant changes and NS means not significant. (A) Control starch diet *ad lib*. (CDA, n = 4 for each data point) and high sucrose diet *ad lib*. (HSDA, n = 3 for each data point). (B) Control starch diet *ad lib*. (CDA, n = 4 for each data point) and control starch diet time-restricted feeding (CDR, n = 4 for each data point). (C) High sucrose diet *ad lib*. (HSDA, n = 3 for each data point) and high sucrose diet time-restricted feeding (HSDR, n = 3 for each data point).

Body temperature variations, every 90 min, in the final two days of the regimen are displayed in Fig 3. Two-way repeated measures ANOVA of body temperature were performed between every two groups (CDA vs. CDR, CDA vs. HSDA, CDR vs. HSDR, HSDA vs. HSDR), and among 4 groups. However, in any analysis, the results showed only a time-effect, without any diet-effect, timing-effect or interaction effect (data not shown). Then JTK_CYCLE analysis determined the phases and amplitudes of each rat under a period of 24 hr (S2 Table). Two-way ANOVA showed that amplitudes were increased in HSD groups, and phases were delayed by time-restricted feeding regime (Fig 3, S2 Table). It is notable that time-restricted feeding rats had delayed elevation of body temperature at the onset of dark period, compared to those in other groups (Fig 3C).

### Blood parameters and hepatic lipids

**Fig 4:**
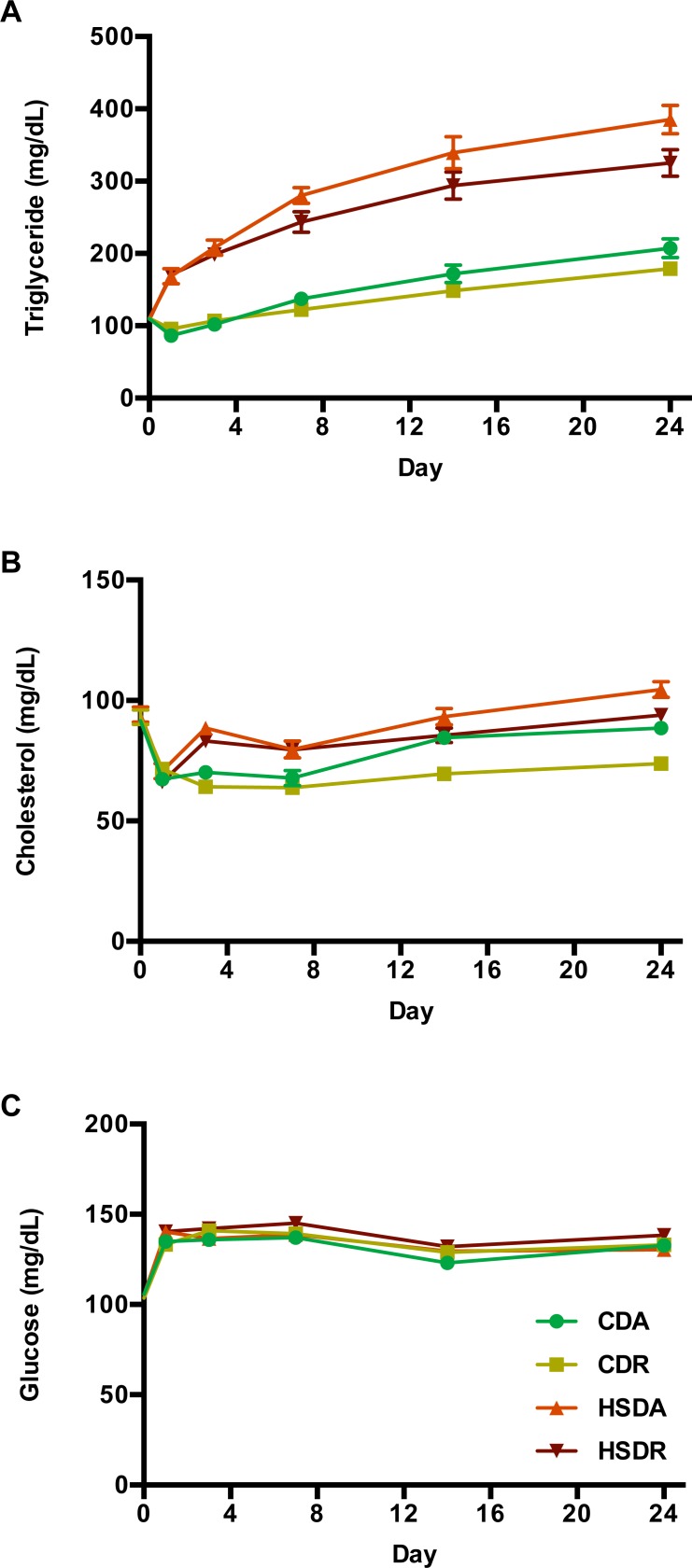
Time-restricted feeding regimen attenuated HSD-induced increase of plasma lipids. Plasma (collected at ZT5 after 4 hr fasting) triglycerides (A), cholesterol (B) and glucose (C) variations measured at day 0, 1, 3, 7, 14 and 24 are shown. Values are means ± SEM, n = 20. Statistical results of two-way repeated measures ANOVA are indicated in S3 Table. 0.05 represents for significant changes and NS means not significant.

To determine whether time-restricted feeding of HSD prevents hyperlipidemia in rats, plasma triglyceride and cholesterol levels were measured. Plasma triglycerides levels were found to be continuously elevated over the subsequent four weeks in all rats, and were significantly increased by HSD compared to that in CDR rats (Fig 4A and S3 Table). However, although time-restricted feeding of HSD (HSDR) clearly attenuated the increase of triglycerides, they were maintained at higher levels compared to that in CD groups (Fig 4A and S3 Table). The total cholesterol concentration in HSD groups was elevated, as seen in Fig 4B. While time-restricted feeding of CD significantly lowered the cholesterol levels, HSDR rats showed no significant decrease (Fig 4B and S3 Table). Plasma glucose levels were not influenced by HSD, fed either *ad lib*. or in a time-restricted manner, over the entire experimental period (Fig 4C and S3 Table).

**Fig 5:**
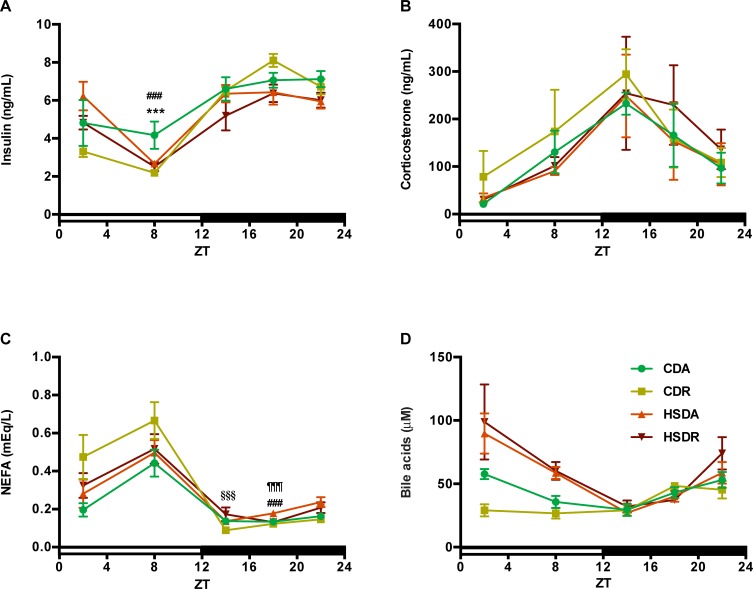
Blood parameters including insulin, corticosterone, non-esterified fatty acid and bile acids, changed little under HSD. Diurnal variations of blood insulin (A), corticosterone (B), non-esterified fatty acid (NEFA) (C) and bile acids (D) in the end of experiment are shown here. Values are means ± SEM, n = 4 at each data point. Statistical results of two-way ANOVA are indicated in S4 Table. 0.05 represents for significant changes and NS means not significant. When the interaction is significant, Student’s *t* test using the residual mean square was performed: *** indicated the values differed significantly (p<0.001) between CDA and CDR groups; ^###^ indicated the values differed significantly (p<0.001) between CDA and HSDA groups; ^§§§^ indicated the values differed significantly (p<0.001) between CDR and HSDR groups; ^¶¶¶^ indicated the values differed significantly (p<0.001) between HSAD and HSDR groups.

Next, we measured the diurnal variations of insulin, corticosterone, non-esterified fatty acid (NEFA), and bile acids. Serum was collected at five different time points (blood was collected when rats sacrificed) until the final day. Insulin responds to carbohydrate-lipid metabolism, and glucocorticoid hormone, corticosterone (cortisol in human), which has a role in circadian oscillations and lipid metabolism [19–22]. Insulin levels were lowered in HSD groups at ZT18 and ZT22. And at ZT8, CDA rats showed higher insulin level than CDR and HSDA ones (Fig 5A). Corticosterone showed no significant difference among the groups (Fig 5B). At ZT18, NEFA was increased by HSD and significantly suppressed in HSDR rats (Fig 5C). HSD significantly increased serum bile acid levels in the inactive phase (light period, at ZT2 and ZT8) (Fig 5D), although time-restricted feeding did not change the HSD-induced bile acid elevation.

**Fig 6:**
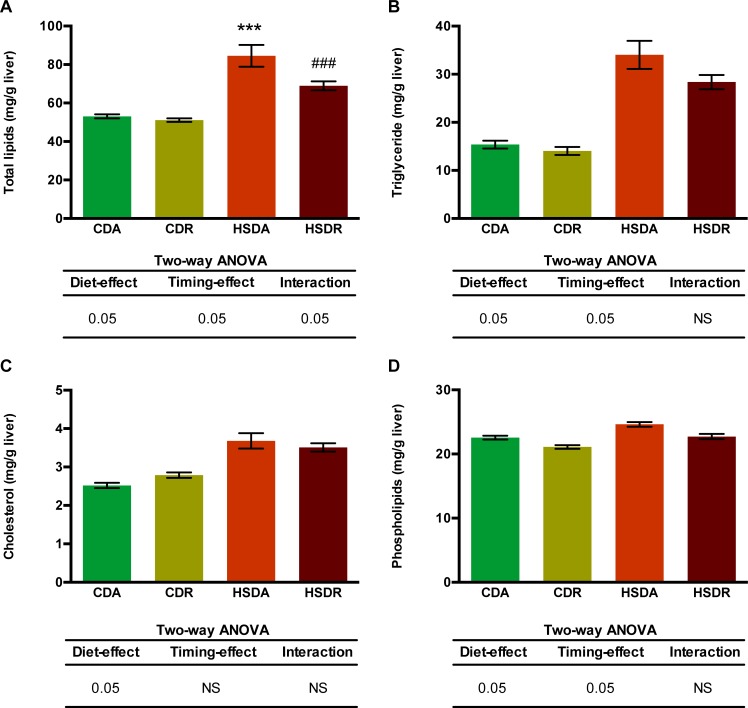
Time-restricted feeding regimen suppressed HSD-induced hepatic lipid accumulation. Liver total lipids (A), triglycerides (B), cholesterol (C) and phospholipids (D) amounts are shown. Values are means ± SEM, n = 20. In the tables of two-way ANOVA results, 0.05 represents for significant changes and NS means not significant. When the interaction is significant, Student’s *t* test using the residual mean square was performed: *** indicated the value differed significantly (p<0.001) from the value of CDA group; ^###^ indicated the value differed significantly (p<0.001) from the value of HSDA group.

We further measured the hepatic lipid contents to determine whether time-restricted feeding regimen would ameliorate HSD-induced hepatic lipid deposition (Fig 6). Liver total lipids were significantly increased in HSD-fed rats, and time-restricted feeding suppressed the elevation (Fig 6A). While hepatic triglycerides and phospholipids were significantly increased by HSD, time-restricted feeding regimen suppressed the elevation (Fig 6B and 6D); however, it did not change the HSD-induced increased cholesterol level (Fig 6C). Although hepatic total lipids were significantly decreased in HSDR rats (Fig 6A), liver weights were not decreased (Table 2). This might be due to increase in other components of liver, such as protein, water, or glycogen. Our results demonstrated that time-restricted feeding of HSD attenuated both blood and hepatic lipid accumulation, resulting from excess sucrose intake.

### Hepatic gene expression

HSD fed *ad lib*. increased lipid accumulation both in blood and liver, and time-restricted feeding regimen amended hyperlipidemia and fatty liver. In order to evaluate whether time-restricted feeding of HSD could contribute to improved gene expression signatures, we measured the expression of hepatic circadian clock genes and lipid metabolism genes that coordinate to maintain lipid homeostasis.

**Fig 7:**
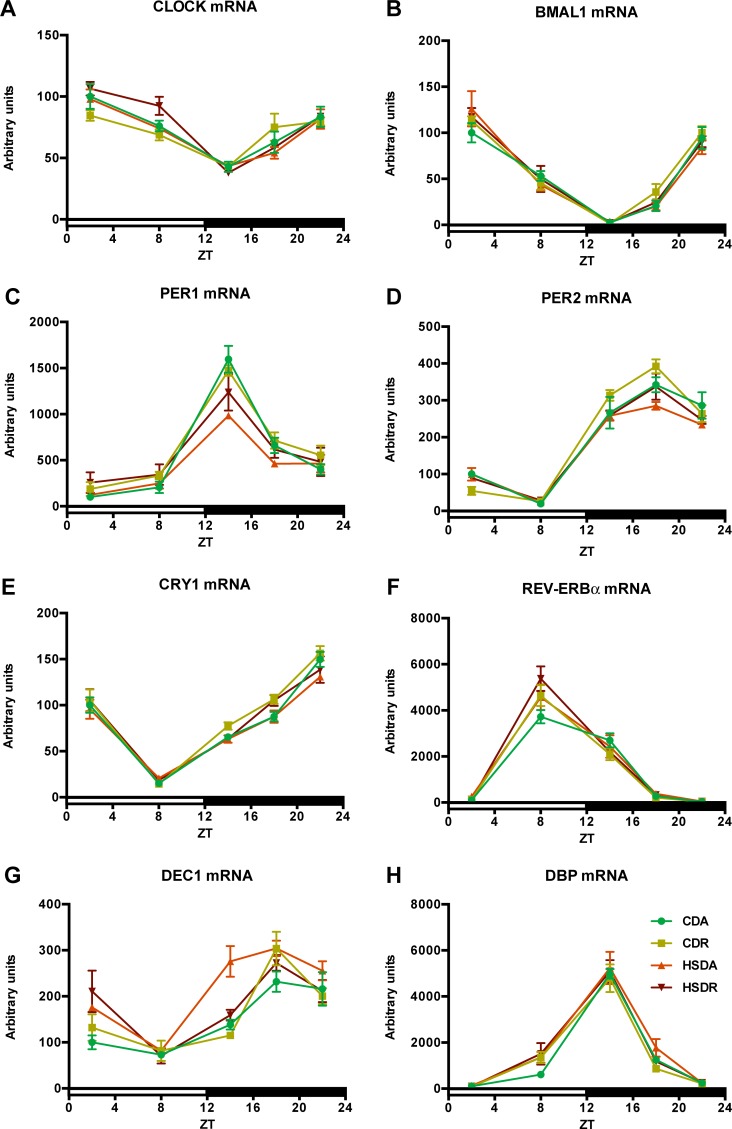
HSD rarely affected liver clock genes expression. Circadian oscillations of clock genes *CLOCK* (A), *BMAL1* (B), *PER1/2* (C and D), *CRY1* (E) *REV-ERBα* (F), *DEC1* (G) and *DBP* (H) are shown as relative to *APOE* mRNA level. Values are means ± SEM, n = 4 at each data point. The average value at ZT2 of each gene in CDA rats was defined as 100. Statistical results of two-way ANOVA are indicated in S5 Table. 0.05 represents for significant changes and NS means not significant.

Circadian clock genes, *CLOCK* (circadian locomotor output cycles protein kaput), *BMAL1* (brain and muscle Arnt-like protein 1), *PER* (period) *1/2*, *DEC* (differentiated embryo chondrocytes) *1/2*, *CRY* (cryptochrome) *1/2*, and *REV-ERB* (nuclear receptor subfamily 1, group D, member 1) *α/β*, also *DBP* (D site of albumin promoter binding protein) encode important transcription factors involved in the regulation of hepatic circadian rhythms and lipid metabolism [23]. The expression or oscillation patterns of these genes were not altered among the groups, except for some minor changes at several time points (Fig 7). Neither HSD nor time-restricted regimens had an influence on rhythmic expression of genes, including phase, period, and amplitude (Fig 7). There was an increase of *DEC1* mRNA at ZT14, induced by HSD and significantly suppressed by HSDR (Fig 7G). Although insulin had been reported to induce the expression of *DEC1* gene [24], we did not observe any corresponding change of insulin concentration in our current study (Fig 5A). These results indicate that HSD itself rarely affected the expression of circadian clock genes and no specific change was observed by time-restricted feeding regimen.

**Fig 8:**
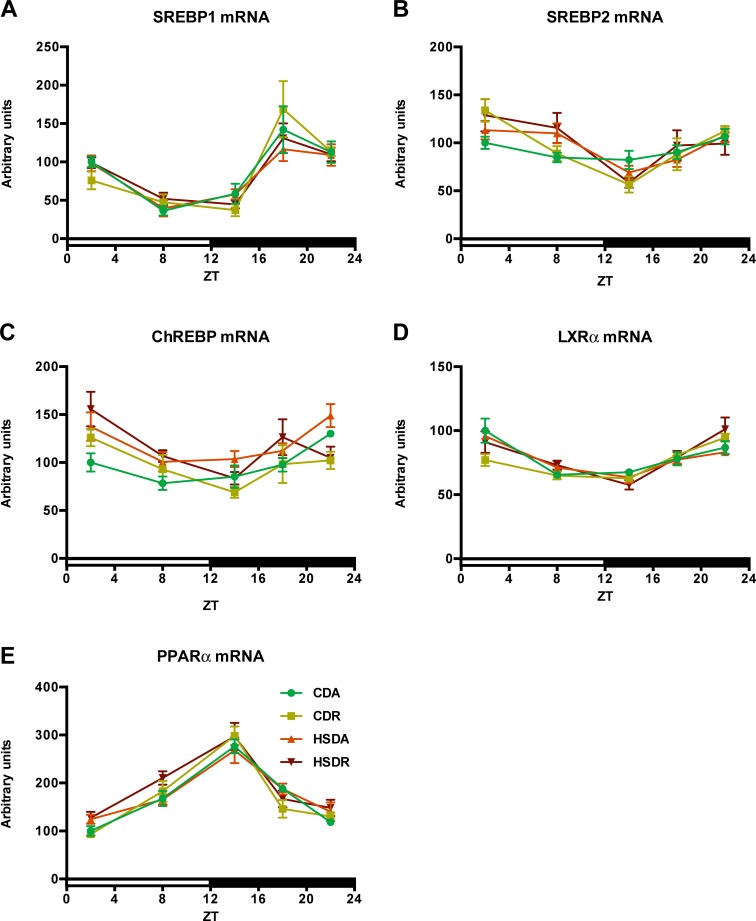
HSD affected little on gene expressions of important transcription factors involved in lipid metabolism. Circadian oscillations of transcription factor genes *SREBP1*/2 (A and B), *ChREBP* (C), *LXRα* (D) and *PPARα* (E) are shown as relative to *APOE* mRNA level. Values are means ± SEM, n = 4 at each data point. The average value at ZT2 of each gene in CDA rats was defined as 100. Statistical results of two-way ANOVA are indicated in S6 Table. 0.05 represents for significant changes and NS means not significant.

Lipid metabolism is known to be mainly governed by several transcription factors, such as *SREBP* (sterol regulatory element binding protein) *1/2*, *ChREBP* (carbohydrate response element binding protein), *LXRα* (nuclear receptor subfamily 1, group H, member 3), and *PPARα* (nuclear receptor subfamily 1, group C, member 1) [25,26], which are also reported to show circadian oscillation [27–29]. We speculated that these transcription factors would also be affected by HSD and time-restricted feeding, since HSD-induced hepatic lipid accumulation was clearly ameliorated by time-restricted feeding regimen (Fig 6). However, expression of these transcription factor genes was hardly affected by HSD or time-restricted feeding, and all the four groups displayed similar expression patterns (Fig 8). This indicated that attenuation of HSD-induced abnormal lipid accumulation in liver and blood by time-restricted feeding regimen is probably independent of the circadian oscillation-transcription factors pathway mediating lipid metabolism.

## Discussion

Metabolic syndrome is currently quite prevalent due to the general change in diet from natural food to processed food containing high fat, sugar, and salt [2]. Excessive sucrose has recently been implicated to play an important role in metabolic syndrome [4,5,30]. Excess sucrose has been reported to induce excessive energy intake-independent obesity and fatty liver in rats, accelerating features of metabolic syndrome [30]. Obesity-induced insulin resistance that causes nonalcoholic fatty liver disease (NAFLD) is a common pathogenic characteristic of metabolic syndrome [31]. NAFLD plays a critical role in development of metabolic syndrome. In our four-week study, HSD dramatically increased lipid accumulation in both blood and liver, indicating pathogenesis of fatty liver. Time-restricted feeding of HSD, only in the active phase, suppressed the increased lipid deposition practically, displaying amelioration of the fatty liver development.

In human and animal studies, sucrose has been reported to decrease insulin sensitivity and induce insulin resistance [3,32–34], which is a contributing factor to obesity. Metabolic syndrome is not always associated with obesity [35,36]; excess sucrose may also contribute to the development of metabolic syndrome without resulting in obesity [32]. In the present study, we propose that HSD-cause fatty liver may be independent of obesity, since there was no increase of body weight in the HSD groups (Table 2). Moreover, we had earlier reported that insulin is a synchronizer for liver clock [37], and plays an essential role in connecting lipid metabolism with circadian oscillations. In this study, insulin was only slightly affected by time-restricted feeding of HSD (Fig 5A). Sucrose has been reported to be addictive, and excessive intake acts on the brain by affecting hormone (e.g. ghrelin and leptin) regulation [35]. We hypothesized that sucrose might have potential effects on hormones, such as corticosterone that we measured in this study. Corticosterone is a glucocorticoid in rodents, higher levels of which were reported in obese rats [38]. Since some reports showed that corticosterone is associated with metabolic diseases [39,40], we speculated that corticosterone would respond to HSD and time-restricted feeding. However, no significant alteration was observed (Fig 5B).

HSD-induced plasma lipid accumulation showed similar alteration pattern as that in the liver, and time-restricted feeding of HSD exhibited amelioration effects on dyslipidemia. Hepatic lipogenesis is known to be directly controlled by the molecular clock [27,29]. Accumulating evidence has indicated that lipid metabolism demonstrates circadian oscillations mainly achieved from collaborative activities of clock genes and key transcription factors controlling lipid metabolism [27,29,41–43]. Disruption of the circadian rhythm in clock gene knockout mice has been reported to be another pathogenic factor of metabolic syndrome [12,44]. Hence, we hypothesized that the connection between circadian oscillation and metabolic state is the mechanism by which time-restricted feeding of HSD suppressed the increased lipid accumulation. However, the core clock feedback loop was slightly altered by HSD or time-restricted feeding (Fig 7). Core clock genes, *CLOCK* and *BMAL1*, which form heterodimers to activate transcription of clock-controlled genes [45], showed no obvious alteration (Fig 7A and 7B). In peripheral tissues, clock genes oscillating in the feedback loop dominate the physiological processes, including glucose and lipid homeostasis, and are sensitive to metabolic signals [27,45]. These clock genes, such as *PER1/2*, *CRY1/2*, and *REV-ERBα/β*, also displayed minor changes in our study (Fig 7C–7H). Moreover, downstream of this core clock loop, transcription factors governing lipid homeostasis (e.g. SREBPs, ChREBP, LXRα, and PPARα) would be the most important regulators of lipid metabolism genes. We, however, did not observe any significant variation (Fig 8), despite the obvious suppression of lipid accumulation by time-restricted feeding of HSD.

Of note, there was a delay of body temperature elevation at the onset of dark period (active phase) in time-restricted feeding rats (Fig 3 and S2 Table). Body temperature usually oscillates between 37–39°C in mammals under the control of the hypothalamus; diurnal variations of body temperatures are associated with energy metabolism oscillations [46]. We speculated that there would be more energy expenditure in HSDR rats, since time-restricted feeding regimen attenuated HSD-induced lipid accumulation. However, HSDR rats showed similar amplitudes of body temperature as HSDA rats. Although there was no significant interaction effect of diet and timing on the phases (S2 Table), HSDR rats had a tendency to delay the body temperature elevation at the onset of dark period (Fig 3C). Although this delay did not explain the suppression of lipid accumulation in blood and liver, the potential role of this delay in lipid metabolism remains to be uncovered.

It is also notable that rats under *ad lib*. access to HSD had less food intake during the inactive phase compared to CD rats (Fig 2). Although it is clearly different from the blunted diurnal feeding rhythm in mice under high-fat diets [13], HSD-fed rats had more robust feeding rhythms than CD-fed rats. Since circadian and feeding rhythms work in harmony to entrain metabolic pathways [11,13,47], the altered feeding rhythm in HSD rats would impact metabolic processes via feeding behavior modulated by the hypothalamus. Hence, feeding behavior or feeding rhythm would have a potential role in ameliorating HSD-induced hyperlipidemia in time-restricted feeding rats. Although a robust feeding rhythm was considered better for the amelioration of metabolic abnormalities [13], the robust feeding behavior induced by HSD might have a role in disturbing lipid metabolism in rats; however, further research is required to explain this phenomenon. Although time-restricted feeding of HSD, within active phase of rats, showed negligible restriction of daily calorie intake, it is important of restriction of food availability during an appropriate period. This effect is not achieved from the mechanism at a gene expression level. The changes at protein level may play roles in achieving this effect. The gut-microbiota system and muscles, connecting absorption, transportation, nutrient utilization, and liver lipid metabolism, may be affected by time-restricted feeding regimen.

Excess sucrose intake has been recognized as the main causative factor in metabolic syndrome [4,5,30]. Time-restricted feeding regimen of HSD, within active phase, is an effective, as well as practical, way to attenuate HSD-induced adverse effects, especially its impact on fatty liver. Time-restricted feeding intervention has been successful in several animals including Zucker obese rats [15], mice [13,14,48], and human [49], with different kinds of diets. Compared to most diet and nutrition interventions, in treatment of diet-induced metabolic diseases, which focus on reducing quantity and restricting energy intake, time-restricted feeding regimens have become a preferable intervention strategy. Our study of HSD time-restricted feeding in Wistar rats within 12 hr active phase also establishes a new evidence of achievement of this time-restricted feeding regimen.

## Supporting information

S1 Table Primer sequences for quantitative real-time PCR. (XLSX)

S2 Table Rhythmic analysis of body temperature by JTK_cycle analysis in Fig 3. (XLSX)

S3 Table Statistical results of plasma parameters in Fig 4. (XLSX)

S4 Table Statistical results of blood parameters in Fig 5. (XLSX)

S5 Table Statistical results of liver clock gene expression in Fig 7. (XLSX)

S6 Table Statistical results of liver transcription factors gene expression in Fig 8. (XLSX)
